# Therapeutic potential of *Cordyceps militaris* cultivated with *Ginkgo biloba* seeds for alleviating western diet-induced type 2 diabetes and diabetic nephropathy

**DOI:** 10.3389/fphar.2025.1562116

**Published:** 2025-05-29

**Authors:** Shinn-Zong Lin, Wei-Wen Kuo, Bruce Chi-Kang Tsai, Catherine Reena Paul, Chia-Hua Kuo, Dennis Jine-Yuan Hsieh, Shih-Wen Kao, Pei-Ying Pai, Shan-Jun Chen, Chih-Yang Huang, Kuan-Ho Lin

**Affiliations:** ^1^ Bioinnovation Center, Buddhist Tzu Chi Medical Foundation, Hualien, Taiwan; ^2^ Department of Neurosurgery, Hualien Tzu Chi Hospital, Buddhist Tzu Chi Medical Foundation, Hualien, Taiwan; ^3^ Department of Biological Science and Technology, College of Life Sciences, China Medical University, Taichung, Taiwan; ^4^ Ph.D. Program for Biotechnology Industry, China Medical University, Taichung, Taiwan; ^5^ School of Pharmacy, China Medical University, Taichung, Taiwan; ^6^ Cardiovascular and Mitochondrial Related Disease Research Center, Hualien Tzu Chi Hospital, Buddhist Tzu Chi Medical Foundation, Hualien, Taiwan; ^7^ Laboratory of Exercise Biochemistry, University of Taipei, Taipei, Taiwan; ^8^ School of Physical Education and Sports Science, Soochow University, Suzhou, China; ^9^ Department of Medical Laboratory and Biotechnology, Chung Shan Medical University, Taichung, Taiwan; ^10^ Clinical Laboratory, Chung Shan Medical University Hospital, Taichung, Taiwan; ^11^ Department of Orthopedic Surgery, Chung Shan Medical University Hospital, Taichung, Taiwan; ^12^ School of Medicine, Chung Shan Medical University, Taichung, Taiwan; ^13^ School of Medicine, College of Medicine, China Medical University, Taichung, Taiwan; ^14^ Division of Cardiovascular Medicine, Department of Internal Medicine, China Medical University Hospital, Taichung, Taiwan; ^15^ Home Run Biotechnology Co., Ltd., Tainan, Taiwan; ^16^ Graduate Institute of Biomedical Sciences, China Medical University, Taichung, Taiwan; ^17^ Department of Medical Research, China Medical University Hospital, China Medical University, Taichung, Taiwan; ^18^ Department of Medical Laboratory Science and Biotechnology, Asia University, Taichung, Taiwan; ^19^ Center of General Education, Tzu Chi University, Hualien, Taiwan; ^20^ Department of Emergency Medicine, China Medical University Hospital, Taichung, Taiwan

**Keywords:** diabetes mellitus, diabetic nephropathy, *Cordyceps militaris*, *Ginkgo biloba* seeds, kidney

## Abstract

**Background:**

Diabetic nephropathy (DN), a leading cause of chronic kidney disease and end-stage renal disease, is a serious complication of type 2 diabetes mellitus (T2DM). Current therapies primarily slow disease progression but are unable to reverse kidney damage, highlighting the need for novel therapy to treat DN.

**Objective:**

This study evaluated the therapeutic potential of *Cordyceps militaris* (*C. militaris*) cultivated on *Ginkgo biloba* (*G. biloba*) seeds in ameliorating T2DM and its complications, especially DN. A T2DM mouse model was established using *ApoE* knockout mice fed a Western diet (WD).

**Results:**

Treatment with the specially cultivated *C. militaris* ameliorated hyperglycemia, dyslipidemia and hepatic dysfunction, while mitigating T2DM-induced renal damage. Key biochemical markers, including blood glucose, triglycerides, cholesterol, blood urea nitrogen (BUN), and creatinine, were significantly improved after treatment. Histopathologic analysis revealed restored renal morphology, reduced fibrosis and decreased amyloid deposition. Mechanistic studies showed downregulation of fibrosis-related proteins such as α-SMA, COL1, TIMP-1, CTGF, TGFβ1 and fibronectin, and upregulation of E-cadherin, Smad7 and Klotho, proteins with anti-fibrotic and renoprotective properties.

**Conclusion:**

These results suggest that the specially cultivated *C. militaris* enhances metabolic regulation and renal repair mechanisms, effectively attenuating T2DM-induced renal damage. This unique cultivation approach enriches the bioactive properties of *C. militaris* and offers a promising natural therapeutic strategy for T2DM and DN. Further studies are needed to validate these results in clinical settings and to explore long-term efficacy and safety.

## 1 Introduction

Diabetes mellitus (DM) is a common and serious metabolic disorder that poses a significant challenge to modern healthcare systems. The global prevalence of DM has increased significantly in recent decades. According to the classification system established by the American Diabetes Association, there are two popular types of DM. Type 1 diabetes mellitus (T1DM) is an autoimmune disease characterized by the destruction of insulin-producing β-cells in the islets of Langerhans, accounting for approximately 5%–10% of cases. In contrast, type 2 diabetes mellitus (T2DM) is the more common DM, accounting for up to 90% of cases, and is attributed to a lack of insulin secretion, impaired insulin action, or both. The global rise in obesity is closely associated with a significant increase in the incidence of T2DM and is increasingly observed in younger obese individuals (Sagoo and Gnudi, 2020). One of the causes of T2DM is changes in the dietary habits of modern people, such as the Western Diet (WD) (Clemente-Suárez et al., 2023). Prolonged DM is associated with multiple organ failure and microvascular complications, including neuropathy, retinopathy, and nephropathy (Beckman and Creager, 2016). Diabetic nephropathy (DN), also known as diabetic kidney disease, is one of the major complications of DM and a leading cause of chronic kidney disease and end-stage renal disease. T2DM often progresses to DN, which is the leading cause of end-stage renal disease worldwide and is associated with significant morbidity and mortality (Maggiore et al., 2017). However, standard treatments for DN primarily slow its progression, but are unable to halt or reverse the disease. Therefore, the development of novel therapeutic strategies for the effective management of DN has become imperative (Samsu, 2021).

Traditional Chinese medicines and their bioactive metabolites possess protective properties against various stresses and diseases. These properties make them promising candidates for the development of innovative therapeutic approaches to effectively treat DN (Chang et al., 2013; Liu et al., 2020; Chang et al., 2021; Ho et al., 2024). *Cordyceps militaris* (*C. militaris*), a fungus belonging to the Ascomycota, is highly regarded for its medicinal and nutritional benefits. Known in Asia for centuries, *C. militaris* has been used both as a dietary supplement and in traditional medicine. *C. militaris* is a rich source of various bioactive metabolites such as cordycepin, adenosine, carotenoids, pentostatin, polysaccharides, proteins, ergosterol, and myriocin, among others (Zhang et al., 2019; Zeng et al., 2024). Pharmacological research has shown that *C. militaris* offers remarkable therapeutic benefits for a wide range of health problems. These include diseases of the neurological, respiratory, cardiovascular, hepatic, and renal systems (Zhang et al., 2019). For the treatment of DM and DN, *C. militaris* has been shown to modulate the gut microbiota by enhancing beneficial bacteria and regulating metabolites and metabolic pathways, thereby ameliorating T2DM in mice (Liu et al., 2023). Previous research shows that *C. militaris* extract has potent anti-diabetic and renal protective effects, making it a promising candidate for the development of new treatments for diabetes (Dong et al., 2014). In addition, cordycepin from *C. militaris* has ability against DN by reducing oxidative stress, inflammation, and apoptosis in HK-2 cells through the miR-193b-5p/MCL-1 pathway. In a mouse model, cordycepin improved renal function and pathology (Zheng et al., 2023). *C. militaris* polysaccharides are observed to improve renal function and mitigate inflammation and podocyte injury while restoring autophagy in a mouse model of streptozotocin-induced DN (Chen et al., 2019). In addition, *C. militaris* mitigated DN in a mouse model by reducing blood glucose, markers of renal dysfunction, pathological renal changes, and fibrosis-related proteins while improving lipid metabolism (Yu et al., 2016). These studies indicate the potential of *C. militaris* and its metabolites as a natural therapy for DM and diabetic kidney disease.

*Ginkgo biloba* (*G. biloba*), often referred to as the “living fossil,” is a deciduous tree belonging to the genus *Ginkgo* in the family Ginkgoaceae. Native to East Asia, this species has a long history of use in traditional Chinese medicine. It has been used in the treatment of several conditions, including cognitive impairment, respiratory diseases, and gastrointestinal disorders (Liu et al., 2022; Biernacka et al., 2023). Previous studies have highlighted the diverse therapeutic properties of *G. biloba*, including its anti-inflammatory and anticancer activities. In addition, *G. biloba* has been shown to exert protective effects on various physiological systems, such as the nervous and cardiovascular systems. It also plays a role in preventing damage associated with diabetes, particularly in mitigating the development of diabetes-induced cataracts (Belwal et al., 2019). *G. biloba* seeds, which are rich in starch, protein, and ginkgo oil, serve a dual purpose as both a food and a medicinal agent. They show significant potential in combating inflammation, oxidative stress, bacterial infections, tumor progression, and nerve damage (Liu et al., 2022). A study shows that *G. biloba* seeds alleviate hyperglycemia-induced oxidative stress, reduce insulin resistance, enhance antioxidant defenses, and reduce inflammation in pancreatic β-cells, liver, and kidneys in a T2DM mouse model (Jing et al., 2021). However, *G. biloba* seeds contain toxic metabolites that pose potential health risks and require the development and implementation of specific detoxification strategies, especially when used as a food or medicinal agent (Boateng and Yang, 2021; Biernacka et al., 2023).

Although *G. biloba* seeds have the potential to alleviate complications associated with DM, their toxicity limits their widespread applicability and safe use. *C. militaris* shows significant health benefits in the management of DM. The bioactive metabolites in *C. militaris* contribute to improved metabolic regulation, antioxidant protection, and anti-inflammatory effects, making them valuable in alleviating complications associated with DM, particularly DN. However, improving the efficacy of *C. militaris* remains an area of research that warrants further investigation. Therefore, in this study, *G. biloba* seeds were used as a culture medium for *C. militaris* to explore its potential therapeutic effects in the context of DM and DN. The research aimed to evaluate the functional properties of *C. militaris* cultivated on *G. biloba* seeds and its efficacy in alleviating the complications associated with DM. Using a T2DM mouse model, the study demonstrated that this specially cultivated *C. militaris* mitigated hyperglycemia, dyslipidemia, liver dysfunction and renal damage, especially DN. The results highlighted the bioactive properties of the specially cultivated *C. militaris* in improving metabolic health and reversing kidney injury, underscoring its potential as a novel treatment strategy for DM and DN.

## 2 Materials and methods

### 2.1 Materials

All chemicals used in this study were analytical grade. They were purchased primarily from Sigma-Aldrich (St. Louis, MO, United States) and Merck (Darmstadt, Germany). Any exceptions were explicitly mentioned. The original *C. militaris* (Cordycipitaceae; *C. militaris (L.) Link*) was obtained from the Bioresource Collection and Research Center (BCRC34380; Food Industry Research and Development Institute, Hsinchu, Taiwan; Hsinchu, Taiwan). Prof. Tsung-Jung Ho of the Department of Chinese Medicine, Hualien Tzu Chi Hospital, Buddhist Tzu Chi Medical Foundation (Hualien, Taiwan) and the technical staff of HOME RUN Biotechnology Co., Ltd. (Tainan, Taiwan) performed the identification of the specially cultivated *C. militaris*. The specially cultivated *C. militaris* was examined morphologically and its identity was then confirmed using the 2018 (Version III, Chinese Edition) of the Taiwan Herbal Pharmacopoeia. The specially cultivated *C*. *militaris* was also analyzed using HPLC-MS (Protech Technology Enterprise Co., Ltd., Taipei, Taiwan) to identify its primary metabolites (Supplementary Material). The *C. militaris* cultivated on *G. biloba* seeds (Patent No. I687170 in Taiwan and CN112913572B in China) was processed into pills and manufactured by HOME RUN Biotechnology Co., Ltd. (Tainan, Taiwan). The WD diet (D12079B, carbohydrate: 43% kcal, fat: 40% kcal, and protein: 17% kcal) was purchased from Research Diets, Inc. (New Brunswick, NJ, Unites States). A variety of primary antibodies were used for analysis in the current study. These primary antibodies included mouse monoclonal anti-alpha smooth muscle actin (α-SMA; ab7817, Abcam, Cambridge, United Kingdom), rabbit polyclonal anti-collagen I (COL1; GTX20292, GeneTex, Irvine, CA, Unites States), mouse monoclonal anti-fibronectin (sc-8422, Santa Cruz Biotechnology, Santa Cruz, CA, Unites States), mouse monoclonal anti-glyceraldehyde-3-phosphate dehydrogenase (GAPDH; sc-32233, Santa Cruz Biotechnology), anti-connective tissue growth factor (CTGF; sc-365970, Santa Cruz Biotechnology), anti-tissue inhibitor of metalloproteinase-1 (TIMP-1; sc-365905, Santa Cruz Biotechnology), anti-E-cadherin (sc-8426, Santa Cruz Biotechnology), goat polyclonal anti-transforming growth factor β1 (TGFβ1; sc-31609, Santa Cruz Biotechnology), rabbit polyclonal anti-Smad7 (sc-11392, Santa Cruz Biotechnology), rabbit polyclonal anti-Klotho (A12028, Abclonal, Woburn, MA, Unites States), and mouse monoclonal anti-beta-actin (β-actin, sc-47778, Santa Cruz Biotechnology). The corresponding secondary antibodies (sc-2357, sc-2354, and sc-516102) were purchased from Santa Cruz Biotechnology.

### 2.2 Animal experimental design

The Institutional Animal Care and Use Committee (IACUC) of Hualien Tzu Chi Hospital, Hualien, Taiwan, approved the animal experiment (approval number: 113–43). Four-week-old male C57BL/6JNarl mice (n = 9, RMRC11005) and male Apoeem1Narl/Narl mice (*ApoE* KO mice, n = 27, RMRC13302) were obtained from the National Institutes of Applied Research National Center for Biomodels in Taipei, Taiwan (http://www.nlac.org.tw/RMRC/webc/html/data/show.aspx?ix=1&page=1&kw=13302). The *ApoE* KO mice were generated using CRISPR/Cas9 technology. All animals were housed under controlled conditions, maintained at 22°C ± 2°C with a 12-h light-dark cycle and 55% ± 5% relative humidity, and provided with standard rodent chow and water *ad libitum*. Mice were allowed a 2-week acclimation period before the start of experimental procedures. The C57BL/6JNarl mice (n = 9) served as the control group, while the *ApoE* KO mice were randomly divided into three experimental groups (n = 9 per group): *ApoE* KO mice fed a standard diet (*ApoE* KO mice), *ApoE* KO mice fed a WD (*ApoE* KO mice with WD), and *ApoE* KO mice fed a WD and this specially cultivated *C. militaris* (*ApoE* KO mice with WD + *C. militaris*). The experimental period was 16 weeks. Starting at week 8, two *C. militaris* pills (30 mg each, containing 111.3 μg cordycepin and 9.4 μg adenosine) were administered orally every 2 days. Fasting serum glucose was monitored weekly using Accu-Chek^®^ guide test strips and Accu-Chek^®^ guide meter (Fritz Hoffmann-La Roche AG, Basel, Switzerland), and a serum glucose level higher than 200 mg/dL was considered indicative of T2DM (Chiang et al., 2021; Ohno et al., 2022). At the end of 16 weeks, all mice were sacrificed and the organs were harvested for further analysis. The different biochemical indicators in serum were measured by Arkray Automated analyzer for clinical chemistry (SPOTCHEM EZ SP-4 430, Arkray Inc., Kyoto, Japan).

### 2.3 Preparation of paraffin-embedded tissue sections

Kidney tissues from each experimental group were fixed in 10% formalin for 2 weeks. The samples were then dehydrated through a graded series of ethanol solutions and embedded in paraffin. The paraffin-embedded tissue blocks were then sectioned at 4 μm thickness for further analysis. Kidney sections were deparaffinized with xylene and rehydrated through a graded series of ethanol solutions for further staining (Tsai et al., 2020a; Tsai et al., 2020b).

### 2.4 Hematoxylin and eosin staining

The rehydrated kidney sections were sequentially stained with hematoxylin and eosin followed by rinsing in water. The slides were then dehydrated through a graded ethanol series and immersed twice in xylene. Images of the stained sections were captured using an OLYMPUS BX53 microscope (Olympus^®^ Corporation, Shinjuku-ku, Tokyo, Japan) (Lin et al., 2021).

### 2.5 Masson’s trichrome staining

The rehydrated kidney sections were stained with Masson’s trichrome dye for 5 min and then rinsed with water. After staining, the slides were dehydrated through a graded ethanol series and immersed twice in xylene. Images of stained tissues were captured using an OLYMPUS BX53 microscope (Olympus^®^ Corporation) (Lai et al., 2023).

### 2.6 Picrosirius red staining

Rehydrated kidney tissue sections were stained for collagen type I and type III fibers using the Picrosirius Red Stain Kit (ab150681, Abcam) according to the manufacturer’s protocol. After staining, the sections were thoroughly rinsed to remove excess dye. High-resolution images of the stained tissues were captured using an OLYMPUS BX53 microscope (Olympus^®^ Corporation) (Lattouf et al., 2014).

### 2.7 Immunohistochemical staining

Rehydrated kidney tissue sections were treated with a permeabilization solution and blocking buffer to reduce nonspecific binding, followed by thorough washing with PBS. The sections were incubated with the primary antibody, anti-α-SMA (diluted in 1% horse serum), for 1 h to target specific protein expression. After further washing with PBS, the slides were processed using a horseradish peroxidase-conjugated avidin-biotin complex (ABC) from the Vectastain Elite ABC Kit (Vector Laboratories, Burlingame, CA). The chromogenic detection was performed using NovaRed substrate (Vector Laboratories, Burlingame, CA), and slides were counterstained with hematoxylin for nuclei visualization. Finally, photomicrographs of the stained sections were captured using an OLYMPUS BX53 microscope (Olympus^®^ Corporation) (Tsai et al., 2020a; Tsai et al., 2020b).

### 2.8 Toluidine blue staining

Rehydrated kidney tissue sections were stained with toluidine blue solution (ScyTek Laboratories, West Logan, UT, Unites States) according to the manufacturer’s protocol. Upon completion of the staining process, the sections were thoroughly rinsed to remove excess dye. High-resolution images of the stained tissues were captured using an OLYMPUS BX53 microscope (Olympus^®^ Corporation) (Niculae et al., 2017).

### 2.9 Tissue protein extraction and western blotting

Kidney tissues from each experimental group were homogenized in lysis buffer containing 50 mM Tris-HCl pH 7.4, 150 mM sodium chloride, 1 mM ethylenediaminetetraacetic acid, 1% nonylphenoxypolyethoxylethanol, 0.25% deoxycholic acid, and supplemented with phosphatase inhibitor cocktail 2 (Cat# P5726, Sigma-Aldrich) and protease inhibitor cocktail (Cat# S8830, Sigma-Aldrich) at a ratio of 100 mg tissue per 1 mL lysis buffer. Homogenized samples were stored at −80°C overnight and then centrifuged at 10,000 g for 30 min at 4°C. The supernatant was collected and protein concentrations were determined using the Lowry protein assay. Calculations were performed using Microsoft Excel (Microsoft Corporation, Redmond, WA, Unites States). Protein aliquots were prepared by mixing with 5× loading dye and then heated at 95°C for 5 min. Equal amounts of protein from each sample were separated by 8%, 10%, or 12% sodium dodecyl sulfate-polyacrylamide gel electrophoresis and transferred to 0.45 µm pore size polyvinylidene difluoride (PVDF) membranes (GE Healthcare UK, Ltd., Amersham, United Kingdom). Membranes were blocked in a buffer containing 5% skim milk in TBST buffer (20 mM Tris-HCl, pH 7.6, 150 mM sodium chloride, and 0.1% polysorbate 20) for 1 h at 25°C to prevent non-specific binding. After blocking, the membranes were incubated overnight at 4°C with primary antibodies diluted in blocking buffer. The next day, the membranes were incubated with horseradish peroxidase-conjugated secondary antibodies, and protein bands were visualized using an Image Bright 1,500 imaging system (Thermo Fisher Scientific, Waltham, MA, Unites States) with Immobilon Western chemiluminescent horseradish peroxidase substrate (WBKLS0500, Merck Millipore, Burlington, MA, Unites States) (Lin et al., 2023; Lin et al., 2024). GAPDH or β-actin were used as internal control proteins.

### 2.10 Statistical analysis

Statistical analyses were performed using GraphPad Prism software (version 6.0, CA, Unites States). Data are presented as the mean ± standard deviation (SD) of independent experiments. One-way analysis of variance (ANOVA) followed by Tukey’s *post hoc* test was used to assess the statistical significance of differences between group means. A *p-*value of less than 0.05 was considered statistically significant, whereas *p-*values of less than 0.01 and 0.001 were considered highly significant (*p* < 0.01 and *p* < 0.001, respectively).

## 3 Results

### 3.1 Beneficial effects of the specially cultivated *C. militaris* on mice with T2DM

**FIGURE 1:**
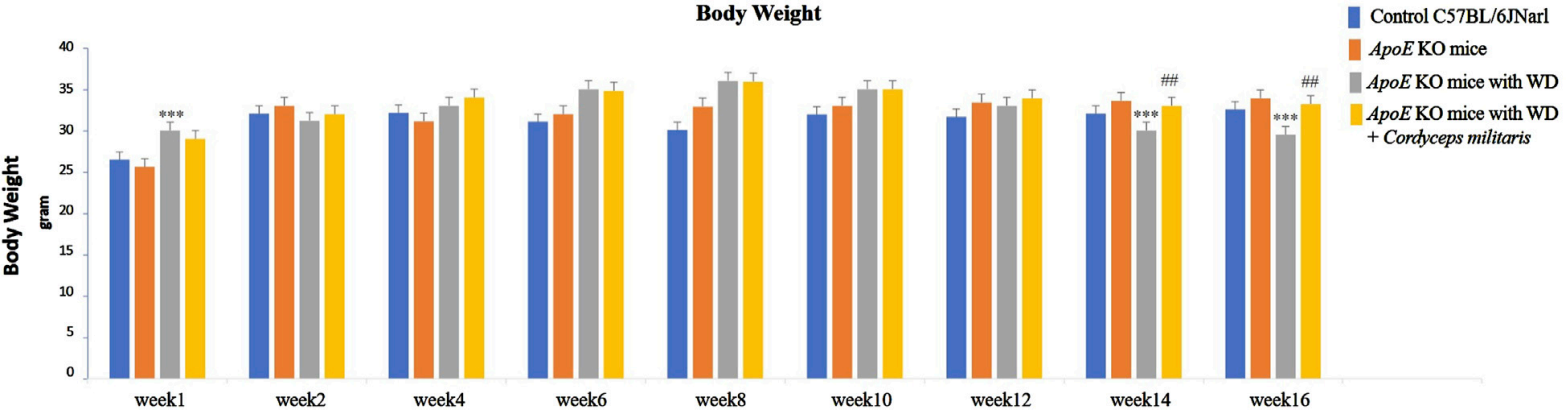
Changes in body weight of *ApoE* KO mice under different experimental conditions. The group of *ApoE* KO mice fed a Western diet (WD) without treatment showed an initial increase in body weight followed by a decrease after 14 weeks. In contrast, *ApoE* KO mice fed a WD and treated with this specially cultivated *Cordyceps militaris* from week 8 maintained a stable body weight throughout the experimental period. Data are expressed as mean ± SD. *** = *p* < 0.001 compared to *ApoE* KO mice fed a standard diet group; ## = *p* < 0.01 compared to *ApoE* KO mice fed with WD without any treatment.

**FIGURE 2:**
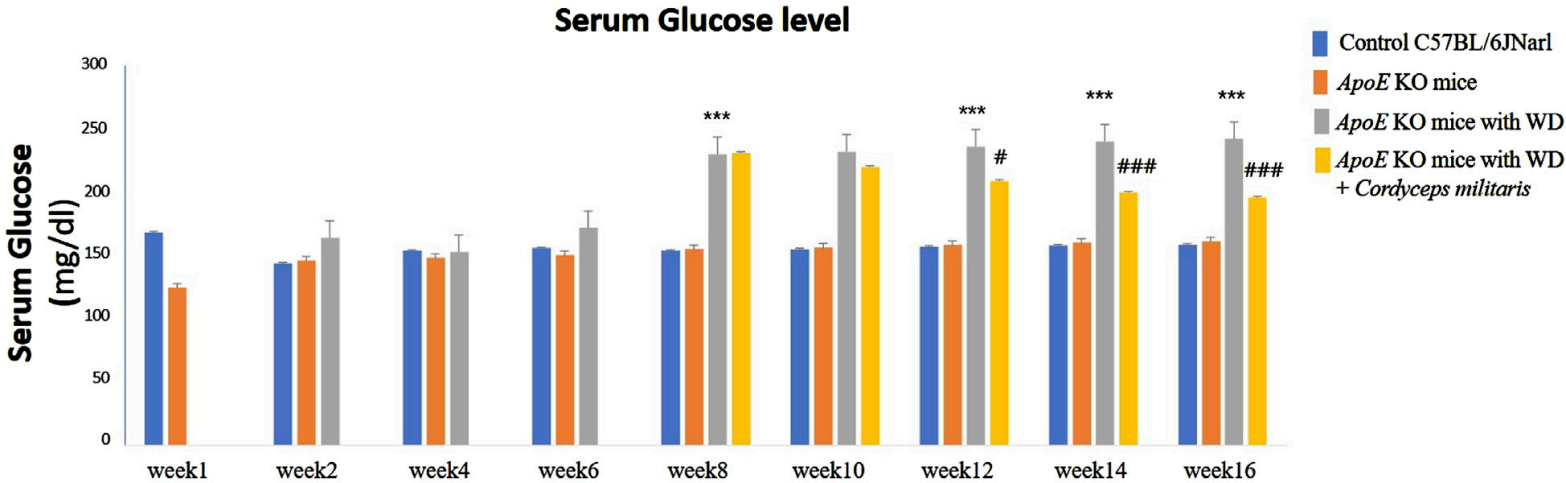
Serum glucose levels in *ApoE* KO mice under different experimental conditions. The *ApoE* KO mice fed a Western diet (WD) alone showed a significant increase in serum glucose levels by week 8. After initiation of this specially cultivated *Cordyceps militaris* treatment at week 8, a progressive decrease in serum glucose levels was observed, which continued consistently until week 16. Data were expressed as mean ± SD. *** = *p* < 0.001 compared to *ApoE* KO mice fed a standard diet group; ### = *p* < 0.001, # = *p* < 0.05 compared to *ApoE* KO mice fed with WD without any treatment.

First, to evaluate the therapeutic potential of this specially cultivated *C. militaris* in the treatment of T2DM, experiments were conducted using an established T2DM mouse model to evaluate the effect of *C. militaris* on body weight and serum glucose levels in *ApoE* KO mice fed a WD over a 16-week period. The results showed significant differences in body weight between the experimental groups. In the group of *ApoE* KO mice receiving a WD without any treatment, a significant increase in body weight was observed during the early weeks of the study. However, this trend was reversed and body weight began to decrease after 14 weeks in the WD group (Figure 1). Conversely, the group of *ApoE* KO mice that received WD and *C. militaris* treatment starting at week 8 showed stable body weight throughout the experimental period (Figure 1). In addition to body weight, the study also monitored serum glucose levels, a critical biomarker in T2DM. Mice exposed to WD alone showed a significant increase in serum glucose levels by week 8 of the study (Figure 2). Treatment with this specially cultivated *C. militaris* began at this week, and a significant reduction in serum glucose levels was observed after 4 weeks of supplementation. This downward trend in serum glucose levels continued consistently through week 16 in the group treated with both WD and this specially cultivated *C. militaris* (Figure 2). These results indicate the potential of this specially cultivated *C. militaris* as a therapeutic agent in the management of DM. Its ability to attenuate hyperglycemia in a T2DM mouse model highlighted its feasibility for further investigation in the treatment of DM.

### 3.2 The specially cultivated *C. militaris* alleviated dyslipidemia and hepatic dysfunction in a T2DM mouse model

**FIGURE 3:**
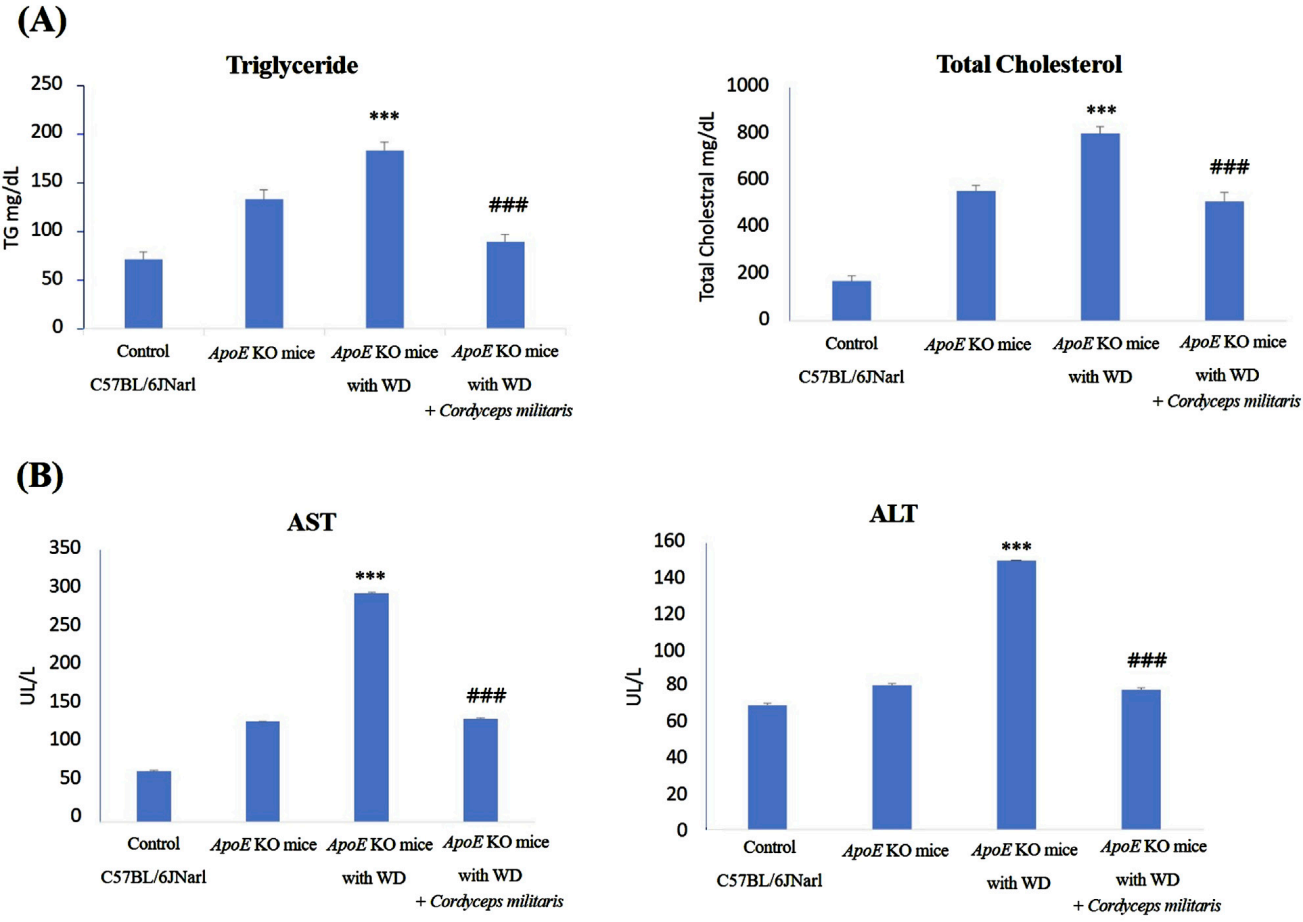
Biochemical analysis of blood lipid levels and liver function in *ApoE* KO mice under different experimental conditions. **(A)** The *ApoE* KO mice fed a Western diet (WD) showed significantly elevated levels of triglycerides and total cholesterol, which were reduced after this specially cultivated *Cordyceps militaris* treatment. **(B)** Elevated serum concentrations of aspartate aminotransferase (AST) and alanine aminotransferase (ALT), markers of liver dysfunction induced by WD, were significantly reduced in the *Cordyceps militaris*-treated group. Data were expressed as mean ± SD. *** = *p* < 0.001 compared to *ApoE* KO mice fed a standard diet group; ### = *p* < 0.001 compared to *ApoE* KO mice fed with WD without any treatment.

Next, to evaluate the potential therapeutic effects of this specially cultivated *C. militaris* on metabolic health, a series of biochemical analyses were performed in a T2DM mouse model. The present study used *ApoE* KO mice fed WD to mimic the metabolic disturbances of T2DM. The results showed that WD significantly elevated blood lipid levels, as evidenced by marked increases in triglyceride and total cholesterol levels (Figure 3A). In addition, WD resulted in significant liver dysfunction as evidenced by elevated serum concentrations of aspartate aminotransferase (AST) and alanine aminotransferase (ALT) (Figure 3B), two well-established markers of liver injury. Interestingly, when the *ApoE* KO mice with WD received this specially cultivated *C. militaris* treatment, a marked improvement in these pathological markers was observed. Specifically, triglyceride and total cholesterol levels were significantly reduced in the *C. militaris*-treated group compared to the WD-only group (Figure 3A). Similarly, the elevated levels of AST and ALT, which are indicative of liver injury, were also significantly reduced after *C. militaris* administration (Figure 3B). These findings suggest that the metabolic abnormalities induced by T2DM, including dyslipidemia and liver dysfunction, were ameliorated. It suggested that this specially cultivated *C. militaris* appeared to exert a protective effect by ameliorating lipid abnormalities and reducing liver damage induced by T2DM.

### 3.3 *C. militaris* reduced renal dysfunction in a mouse model of T2DM

**FIGURE 4:**
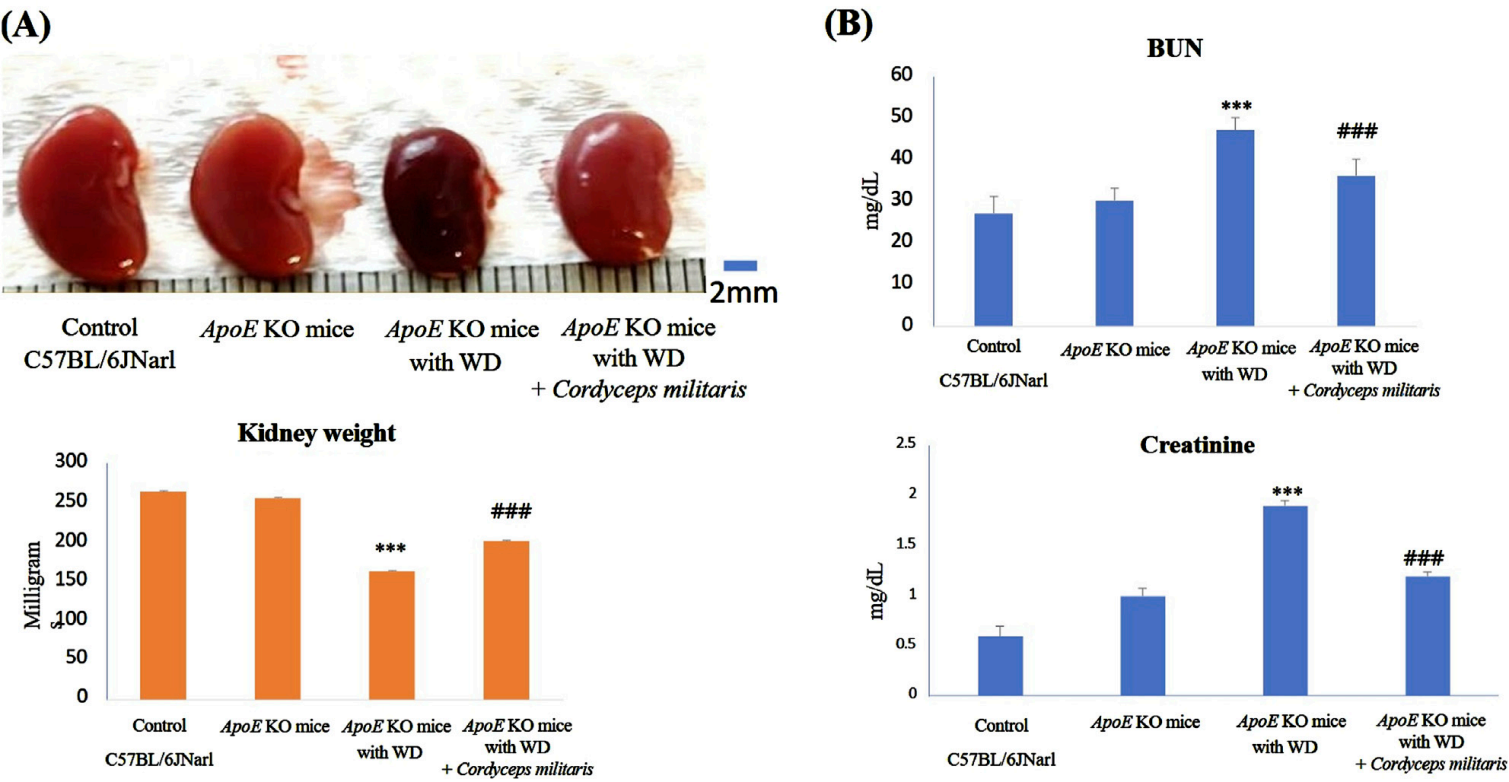
Evaluation of renal dysfunction and histopathologic changes in *ApoE* KO mice under different experimental conditions. **(A)** The *ApoE* KO mice fed a Western diet (WD) showed changes in renal morphology and a decrease in renal weight. **(B)** The *ApoE* KO mice showed significantly elevated blood urea nitrogen (BUN) and serum creatinine levels, indicating renal dysfunction. However, this specially cultivated *Cordyceps militaris* treatment improved the kidney and significantly decreased the BUN and serum creatinine levels. Data were expressed as mean ± SD. *** = P < 0.001 compared to *ApoE* KO mice fed a standard diet group; ### = P < 0.001 compared to *ApoE* KO mice with WD without any treatment.

**FIGURE 5:**
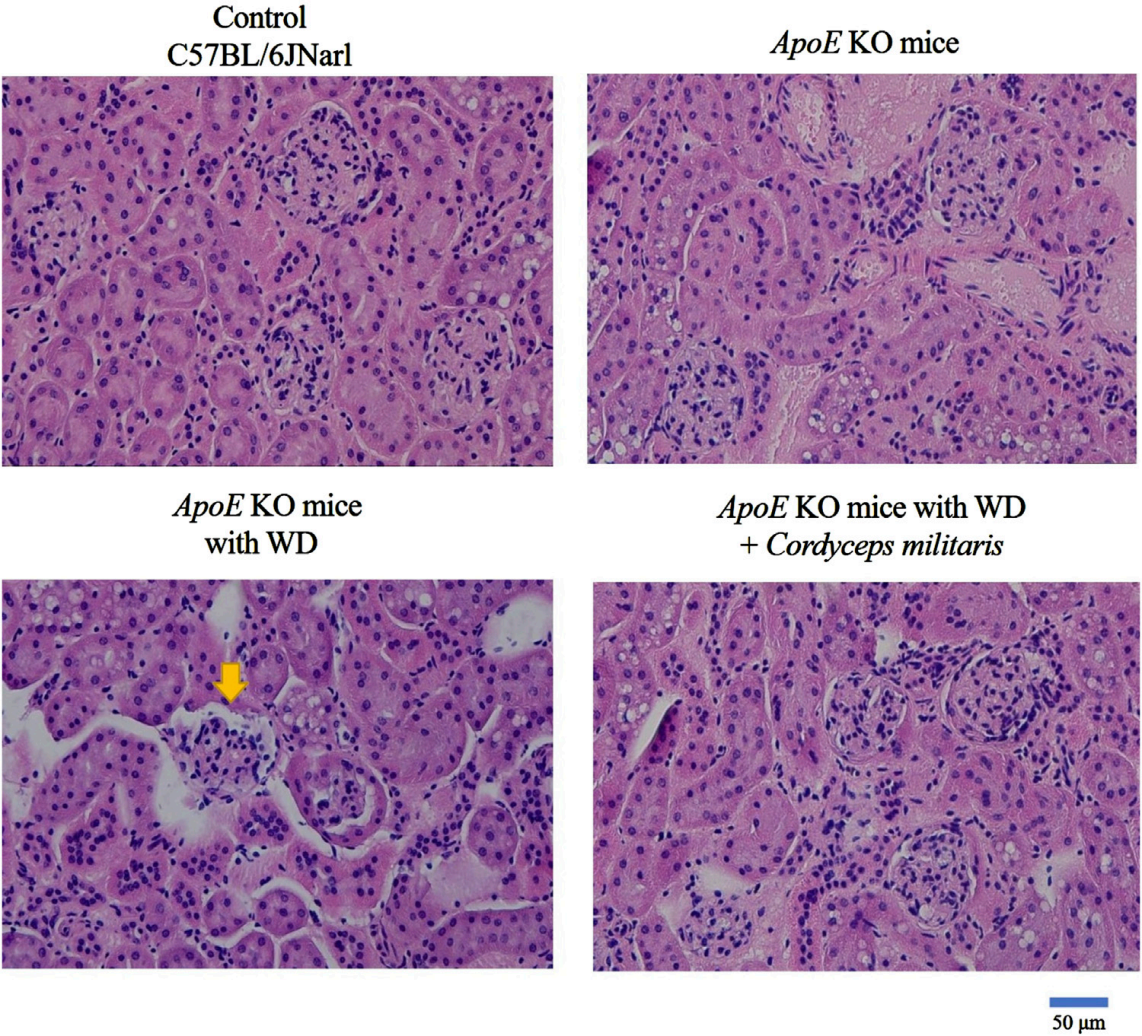
Restoration of renal glomerular morphology by *Cordyceps militaris* treatment in *ApoE* KO mice. Histopathological analysis using Hematoxylin and eosin staining showed severe morphological abnormalities in renal glomeruli, including shrinkage and atrophy, in WD-fed mice. These pathological changes were ameliorated in the *Cordyceps militaris*-treated group and the glomerular morphology resembled that of healthy controls.

DN is recognized as one of the primary and most serious complications of T2DM, often leading to progressive renal dysfunction. To evaluate the beneficial effects of this specially cultivated *C. militaris* on renal dysfunction in T2DM, various indicators of renal health were evaluated in this study. The values such as kidney weight, BUN and serum creatinine levels were measured to determine the extent of renal damage and the therapeutic potential of this specially cultivated *C. militaris*. The results showed that the fibrosis morphologies, including dark brown and shrinking morphology, were observed in *ApoE* KO mice fed with WD. Similarly, a significant reduction in kidney weight was observed in *ApoE* KO mice fed with WD, accompanied by markedly elevated levels of BUN and serum creatinine. (Figures 4A,B). Because glomerular abnormalities are the hallmark of DN and lead to structural destruction of the kidney (Kaya et al., 2021), histopathologic analysis of renal glomeruli was performed using Hematoxylin and eosin staining. The stained tissue sections revealed pronounced morphological abnormalities in renal glomeruli, in the WD-fed *ApoE* KO mice (Figure 5). Remarkably, treatment with this specially cultivated *C. militaris* was found to alleviate these pathological changes. In T2DM mice receiving *C. militaris*, kidney weight was improved. Similarly, elevated BUN and serum creatinine levels were significantly reduced (Figure 4). Histological examination further confirmed these results, as the structural integrity of renal glomeruli was visibly restored in *C. militaris*-treated mice, with morphology comparable to that of healthy controls (Figure 5). The data suggest that this specially cultivated *C. militaris* not only improved biochemical markers of renal health, but also facilitated the recovery of renal morphology associated with DN in a T2DM mouse model.

### 3.4 Therapeutic effects of *C. militaris* on diabetes-induced renal fibrosis

**FIGURE 6:**
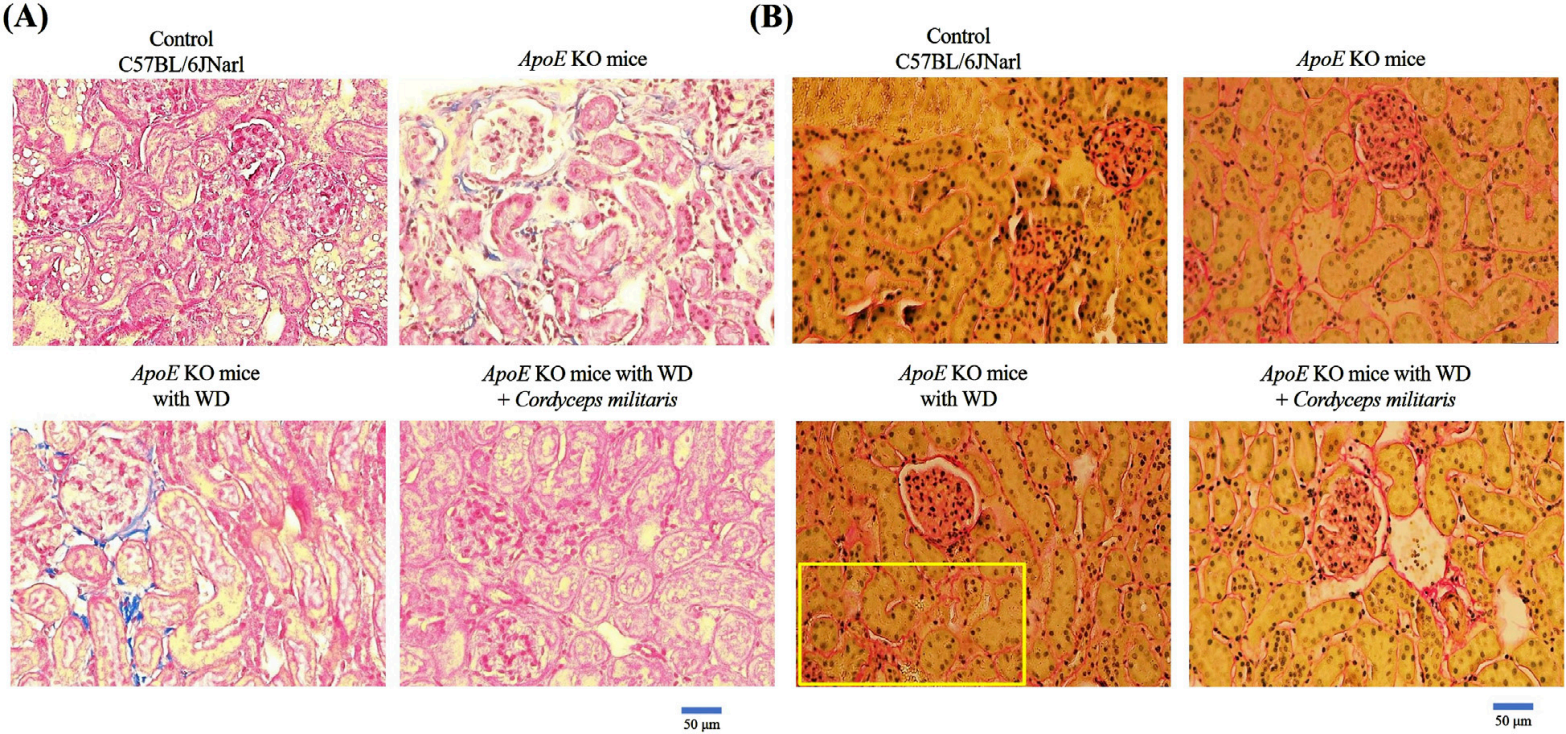
Evaluation of collagen deposition in renal tissue using histological staining. **(A)** Masson’s trichrome staining and **(B)** Picrosirius red staining revealed significant collagen accumulation in the kidneys of *ApoE* KO mice fed a Western diet (WD). This specially cultivated *Cordyceps militaris* treatment remarkably reduced collagen deposition as evidenced by reduced staining intensity and improved tissue morphology.

**FIGURE 7:**
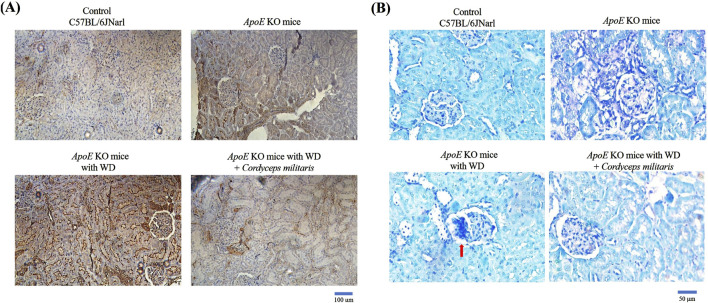
Analysis of fibrosis and amyloid deposition in the kidneys of *ApoE* KO mice. **(A)** Immunohistochemical staining revealed significantly increased expression of α-smooth muscle actin (α-SMA), a marker of fibrosis, in the kidneys of mice fed a Western diet (WD). This specially cultivated *Cordyceps militaris* treatment significantly reduced α-SMA expression, demonstrating its antifibrotic effects. **(B)** Toluidine blue staining showed extensive amyloid deposits in the renal glomeruli of WD-fed mice, which were attenuated after this specially cultivated *Cordyceps militaris* administration, indicating its protective role against amyloid accumulation.

**FIGURE 8:**
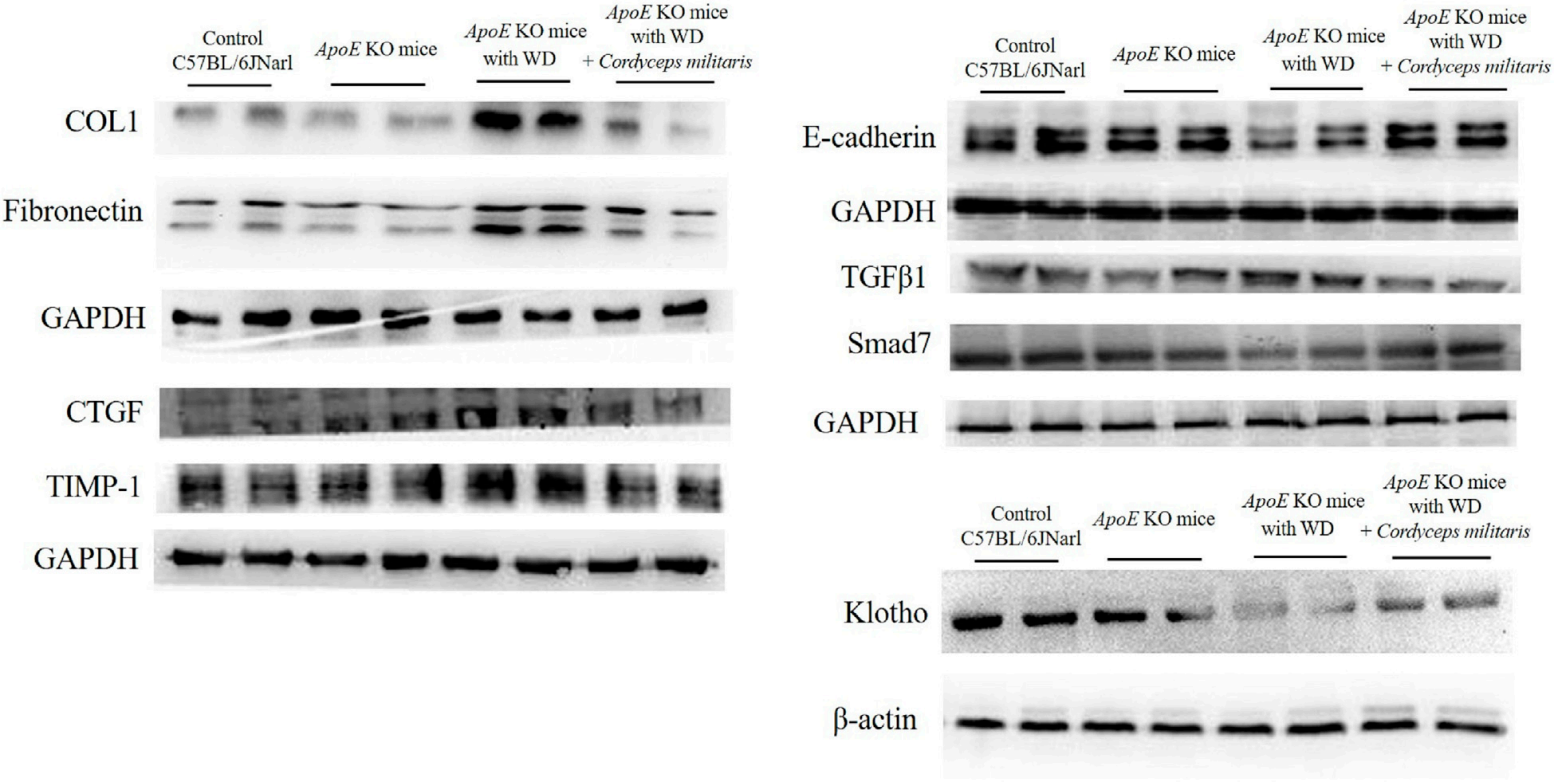
Western blot analysis of fibrosis-related proteins and Klotho expression in the kidneys of *ApoE* KO mice. Western diet (WD)-fed mice showed upregulation of fibrosis-associated proteins, including collagen type I (COL1), fibronectin, connective tissue growth factor (CTGF), tissue inhibitor of metalloproteinase 1 (TIMP-1), and transforming growth factor β1 (TGFβ1), and downregulation of anti-fibrotic proteins E-cadherin and Smad7. This specially cultivated *Cordyceps militaris* treatment reversed these trends by reducing pro-fibrotic proteins, restoring E-cadherin and Smad7 expression, and increasing Klotho level.

Renal fibrosis is recognized as a hallmark pathological feature of DN and contributes significantly to its progression and severity (Xue et al., 2023). In this study, the potential therapeutic effects of this specially cultivated *C. militaris* on DM-induced renal fibrosis were thoroughly investigated by applying various tissue staining techniques and Western blot analysis. Masson’s trichrome staining (Figure 6A, blue area) and picrosirius red staining (Figure 6B, red area) were used to evaluate collagen deposition within the renal tissue. The staining results showed a significant increase in collagen accumulation in the kidneys of WD-fed *ApoE* KO mice. Remarkably, *C. militaris* treatment attenuated this collagen deposition as shown in the staining images (Figure 6). Furthermore, the expression of α-smooth muscle actin (α-SMA), a widely recognized marker of fibrosis, was significantly increased in the kidneys of *ApoE* KO mice fed with WD. However, after *C. militaris* administration, α-SMA levels were significantly reduced, highlighting the potential antifibrotic effects of this specially cultivated *C. militaris* in diabetic kidneys (Figure 7A). To further evaluate the extent of amyloid deposition, toluidine blue staining was performed. Toluidine blue staining revealed pronounced amyloid deposits in the renal glomeruli of WD-fed *ApoE* KO mice. Notably, *C. militaris* treatment attenuated these deposits, suggesting its protective role against amyloid accumulation in the kidneys (Figure 7B). The results of these histologic stains were further supported by Western blot analysis, which detects the expression of key fibrosis-related proteins. In the kidneys of WD-fed *ApoE* KO mice, proteins such as collagen type I (COL1), fibronectin, connective tissue growth factor (CTGF), tissue inhibitor of metalloproteinases (TIMPs), and transforming growth factor β1 (TGFβ1) were upregulated. Conversely, E-cadherin (Chang et al., 2022) and Smad7 (Li et al., 2002), key proteins known for their anti-fibrotic properties, was remarkably downregulated. Importantly, *C. militaris* treatment reversed these pathological trends, demonstrating a reduction in the levels of pro-fibrotic proteins and an upregulation of Smad7 expression (Figure 8). In addition, the study observed that *C. militaris* treatment increased the expression of Klotho, an anti-aging protein with renoprotective properties, which was decreased in the kidneys of WD-fed *ApoE* KO mice (Figure 8). Taken together, the reversal of fibrotic markers, the reduction of amyloid deposition, and the enhancement of protective protein expression highlighted the therapeutic potential of this specially cultivated *C. militaris* in mitigating DM-induced renal fibrosis and kidney damage in DN.

## 4 Discussion

A previous study mentions that *C. militaris* extracts significantly reduce blood glucose, cholesterol, and triglycerides, improve antioxidant capacity, and combat DN by reducing uric acid, creatinine, BUN, and urinary protein levels (Dong et al., 2014). The present study also demonstrates that *C. militaris* cultivated on *G. biloba* seeds has remarkable therapeutic potential by effectively addressing critical complications associated with DM. These included reducing hyperglycemia, alleviating dyslipidemia, and improving liver dysfunction. In addition, it specifically alleviated DN, a serious complication of DM, by reducing renal fibrosis while supporting renal function and metabolic balance. T2DM is a chronic metabolic disorder characterized by insulin resistance and relative insulin deficiency, resulting in elevated blood glucose levels (Galicia-Garcia et al., 2020). The present study demonstrates that this specially cultivated *C. militaris* effectively reduces serum glucose levels in a T2DM mouse model. This finding is consistent with previous research showing the hypoglycemic effects of *C. militaris* (Cheng et al., 2012). The diabetic serum lipid abnormalities commonly observed in T2DM are characterized by an atherogenic lipid profile with high levels of triglycerides, elevated low-density lipoprotein cholesterol, and decreased high-density lipoprotein cholesterol. This lipid imbalance significantly increases the risk of cardiovascular disease in people with DM (Kalra and Raizada, 2024). Moreover, an increase in total cholesterol is associated with a higher risk of DM-related cardiovascular diseases in diabetic patients, whereas a decrease in total cholesterol is associated with a lower risk of cardiovascular diseases (Khil et al., 2023). Non-alcoholic fatty liver disease has a significant and well-documented association with T2DM. It is estimated that fatty liver affects approximately 70%–80% of individuals diagnosed with T2DM, highlighting its high prevalence in this population. Diabetic dyslipidemia contributes significantly to the pathogenesis of fatty liver. The dysregulated lipid metabolism associated with diabetic dyslipidemia leads to excessive lipid accumulation in the liver, thereby promoting the development of hepatic steatosis (Han et al., 2019). Furthermore, associated liver enzymes such as AST and ALT are often significantly elevated in patients with diabetes, reflecting liver dysfunction (Al-Jameil et al., 2014). The current study confirms that WD significantly increases blood lipid levels (triglycerides and cholesterol) and causes liver dysfunction as indicated by elevated serum AST and ALT levels. However, treatment with this specially cultivated *C. militaris* improves these markers by reducing lipid abnormalities and mitigating liver damage. These results highlight the protective effects of *C. militaris* against T2DM-induced metabolic disorders, including dyslipidemia and liver injury. One study shows that patients with diabetic nephropathy have significantly higher levels of total cholesterol and triglycerides than those without nephropathy (Palazhy and Viswanathan, 2017). These findings underscore the link between lipid abnormalities and progression of diabetes-related kidney damage.

DN, the most common complication of DM, is the leading cause of end-stage renal disease in DM patients. Renal fibrosis mediated by TGF-β1 upregulation plays a key role in the onset and progression of diabetic nephropathy by promoting type I collagen synthesis and suppressing its degradation (Cheng et al., 2013; Zhang et al., 2021). Smad proteins are essential components of the downstream signaling pathway involved in TGF-β1-mediated renal fibrosis. While Smad2-4 are activated by TGF-β1 to drive renal fibrosis, Smad7, an inhibitory Smad, counteracts the process of renal fibrosis by suppressing the expression of fibrotic cytokines (Zhang et al., 2021). Additionally, the TGF-β signaling pathway promotes the downregulation of E-cadherin and the expression of mesenchymal markers such as α-SMA and fibronectin, leading to the transformation of injured renal epithelial cells into a mesenchymal-like state. This transition may contribute to increased extracellular matrix production and accumulation (Nagae et al., 2008; Gewin and Zent, 2012). CTGF is a known profibrotic mediator. It is also upregulated in human kidney proximal tubular cells (HKC-8) after TGF-β1 stimulation (Phanish et al., 2006). Additionally, TIMP-1, which inhibits interstitial collagenases and promotes fibrosis progression, is also induced by TGF-β1 stimulation (Zhou et al., 2007; Kim et al., 2018). Together, these studies demonstrate that TGF-β1 plays a central role in the initiation and progression of renal fibrosis. Moreover, Klotho, a protein known for its anti-aging properties, has been shown to be downregulated in T2DM and its associated complication, DN (Tang et al., 2023). In the current study, this specially cultivated *C. militaris* attenuates TGF-β1 expression to reduce fibrotic markers such as α-SMA, COL1, CTGF, TIMP-1 and fibronectin, and increases the expression of anti-fibrotic proteins such as Smad7 and E-cadherin to limit DM-induced renal fibrosis in a T2DM mouse model. Treatment with this specially cultivated *C. militaris* also increases Klotho expression. And then, this specially cultivated *C. militaris* presents ability to restore renal morphology and weight as well as normalize BUN and serum creatinine levels through changes in molecular mechanisms. Furthermore, amyloid plaques are observed in over 70% of patients with T2DM, and these deposits readily develop into mature fibrils (Smith et al., 2022). The kidney is one of the most common organs affected by amyloid deposition (Dember, 2006; Feitosa et al., 2022). The present study shows amyloid deposition in the renal glomeruli of WD-fed *ApoE* KO mice, which was reduced by the specially cultivated *C. militaris* treatment, indicating its potential protective effect in the kidney of T2DM. These findings highlight the therapeutic potential of this specially cultivated *C. militaris* in ameliorating renal injury and fibrosis in DN.

Although this study highlights the therapeutic potential of *C. militaris* cultivated on *G. biloba* seeds in mitigating T2DM and its related complications, it still has some research limitations. In terms of detection indicators, the focus is primarily on a few metabolic indicators, renal function indicators, and fibrosis-related indicators, with a lack of data on other physiological processes such as inflammatory factors and oxidative stress indicators. This limits a comprehensive understanding of the mechanism of action of *C. militaris* in the body. Protein detection is also limited to a few fibrosis-related and anti-fibrosis proteins and does not cover all key proteins involved in renal fibrosis. Regarding the experimental design, the lack of different dosage groups for *C. militaris* hinders the identification of an optimal therapeutic dose, reducing the clinical applicability of the results. Furthermore, the lack of comparative trials with clinical medications makes it difficult to evaluate the relative advantages of this specially cultivated *C. militaris* in clinical application. The study also relies on an animal model that may not fully capture the complexity of DN in humans, thus caution should be exercised in translating these results to clinical practice. In addition, the short duration of the study does not address the long-term safety and efficacy of *C. militaris*. Future research should include clinical trials to validate these findings in humans, explore potential side effects, and optimize dosing regimens. Nevertheless, the results underscore the promising potential of this specially cultivated *C. militaris* as a natural and effective therapeutic strategy for the management of T2DM and its associated complications.

## 5 Conclusion

In conclusion, this study highlighted the therapeutic potential of *C. militaris* cultivated on *G. biloba* seeds in the treatment of T2DM and its complications, especially DN. The results showed that this unique cultivation approach enhanced the bioactive properties of *C. militaris*, enabling it to effectively alleviate hyperglycemia, dyslipidemia, liver dysfunction, and renal damage. In addition, the treatment reduced renal fibrosis while promoting renal repair and metabolic balance. These results highlight the promise of this specially cultivated *C. militaris* as a natural and effective therapeutic option for T2DM and its associated complications, which warrants further investigation and clinical validation.

## Data Availability

The original contributions presented in the study are included in the article/Supplementary Material, further inquiries can be directed to the corresponding authors.

## Abbreviations

ALT, alanine aminotransferase; AST, aspartate aminotransferase; BNU, blood urea nitrogen; C. militaris, Cordyceps militaris; DM, Diabetes mellitus; DN, Diabetic nephropathy; G. biloba, Ginkgo biloba; T2DM, type 2 diabetes mellitus; WD, western diet.
