# Supplementary material for: Therapeutic potential of Cordyceps militaris cultivated with Ginkgo biloba seeds for alleviating western diet-induced type 2 diabetes and diabetic nephropathy
Source: Front Pharmacol. 2025 May 29;16:1562116. doi: 10.3389/fphar.2025.1562116 (PMC12158992; doi:10.3389/fphar.2025.1562116)
Supplement: Supplementary file 1 [file DataSheet1.pdf]

Supplementary Material

HPLC/MS results

Instrument Device :

Instrument Categories	Instrument Model
Pump Model	Agilent 1260 G1312B Binary Pump
AutoSampler	Agilent 1260 G1367E HiP ALS Autosampler
Degasser	Agilent 1260 G1379B Degasser
Column	Phenomenex Kinetex-Phenyl-Hexyl-100A (100 mm x 2.1 mm i.d., 2.6 um)
Mass Spectrometer	AB Sciex Instruments QTRAP 5500
Source type	Turbo V Ion Source

HPLC Method Properties :

Duration : 10 min
Injection volume : 5 ul
Mobile Phase : A: 0.1% (v/v) Formic acid / Water ; B: 0.1% (v/v) Formic acid / Acetonitrile
Step Table :

Step	Total Time(min)	Flow Rate(μl/min)	A (%)	B (%)
0	0.10	100	85.0	15.0
1	2.00	100	85.0	15.0
2	5.00	100	70.0	30.0
3	6.00	100	10.0	90.0
4	7.00	100	10.0	90.0
5	8.00	100	85.0	15.0
6	10.0	100	85.0	15.0

Sample Preparation :

A volume of 100 μL of the sample was taken, followed by the addition of 200 μL of methanol. The mixture was vortexed thoroughly to ensure complete dissolution and subsequently incubated at -20°C for 30 minutes. Centrifugation was performed at 15,000 × g for 10 minutes, and the supernatant was collected and diluted 10× with 50% methanol prior to analysis. (Total dilution factor: 30×)

Mass Spectrometer Information :

Scan Type : MRM (Multiple Reaction Monitor)
Polarity : Positive ion mode
Source temperature : 400 ℃
Data acquisition : Analyst 1.5 software

Parameter Table :

CUR (curtain gas) : 20.00 psi Nebulizing gas (GS1) : 45.00 psi
Collision-activated Dissociation (CAD) : High Heating gas (GS2) : 40.00 psi
Electrospray capillary voltage : 5500.00 V

Preparation of Calibrators :

The powdered standard was dissolved in methanol to prepare stock solutions of each target compound. These stock solutions were then used to prepare mixed working solutions with concentrations around μg/mL, which were stored at -20°C for future use. Calibration solutions were freshly prepared and serially diluted with 50% methanol to achieve a concentration range of ng/mL.

Quantitation Method :

As the provided standards are not isotope-labeled, the Multiple Point External Standard method was employed for quantitation.

Selected Transitions and Parameter Settings

Analytes	Q1 mass	Q3 mass	DP (V)	EP (V)	CE (V)	CXP (V)
Adenosine	268.2	85.2<br>136.2	180	10	34<br>24	13<br>10
Cordycepin	252.2	119.2<br>**136.2**	220	10	57<br>**21**	10<br>**12**

Note: Red indicates the ion pairs used for quantitation.

Calibration Curve of Standard

Linear regression **Weighting：1/x**

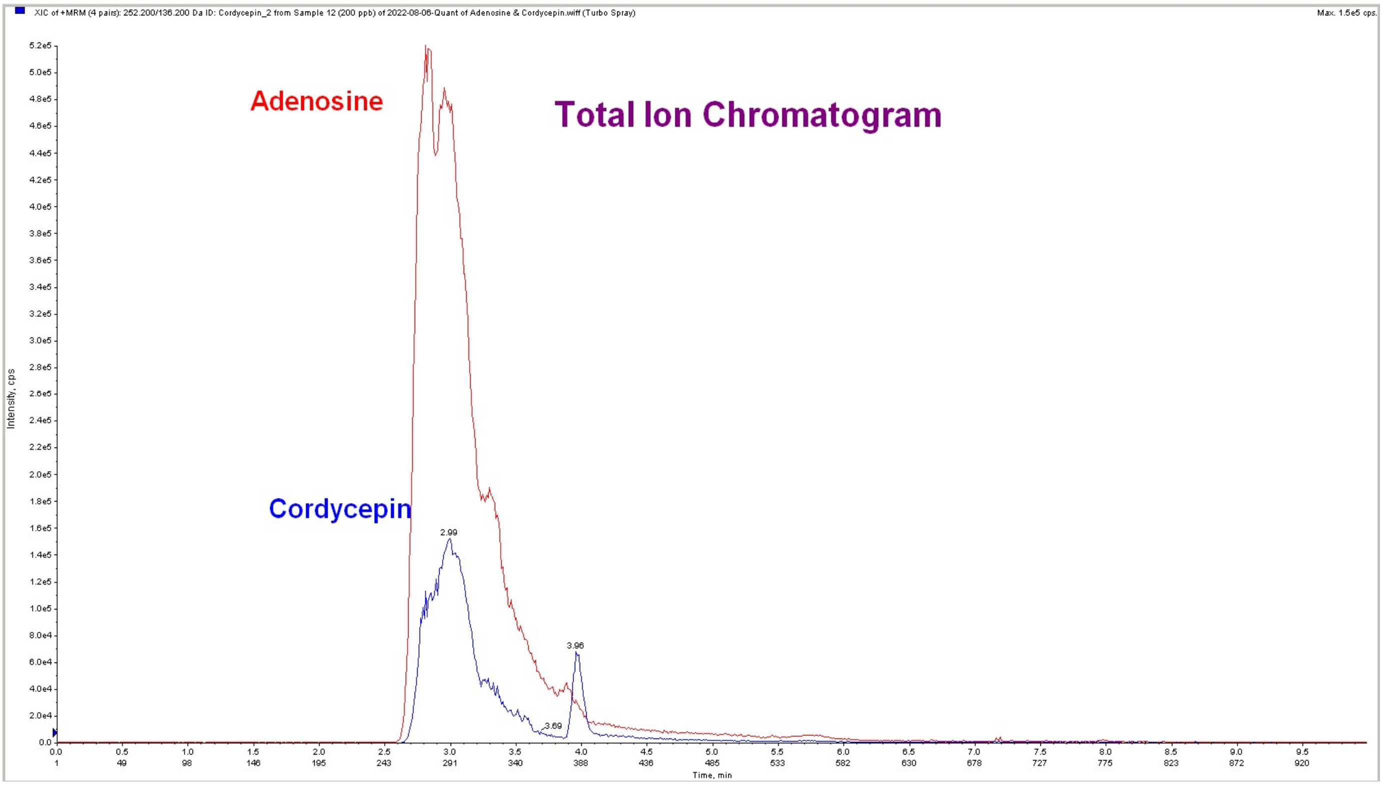

Note: Since cordycepin is a derivative of adenosine, their physicochemical properties are similar, making it difficult to completely separate them under HPLC conditions. However, the EIC chromatogram shows that the selected ion pairs produce distinct signals at their respective retention times, indicating specificity.

Sample Spectrum

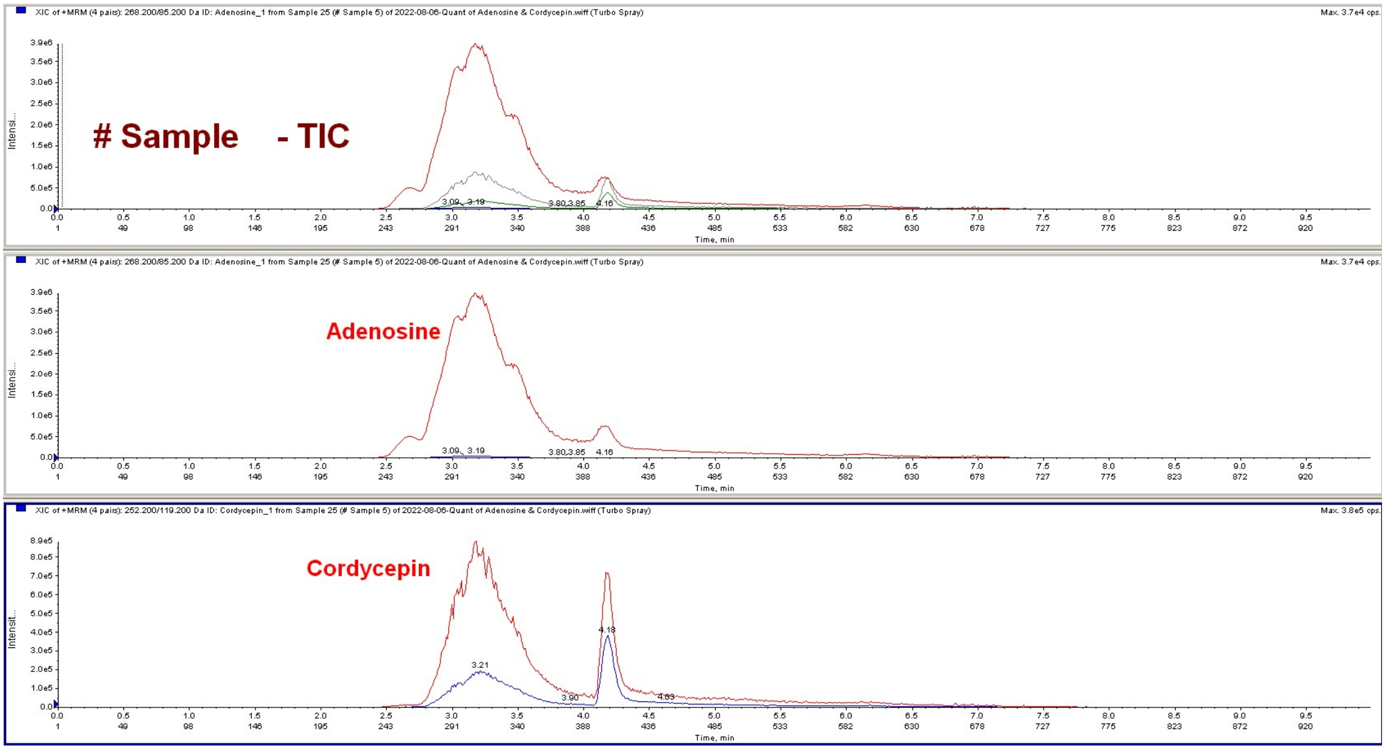

Analyte Name: Adenosine

Regression Equation: $y = 6.56e{+}004\ x + 3.87e{+}004\ (r = 0.9999)$

Expected Concentration (ng/ml)	Mean Calculated Concentration (ng/ml)	% Accuracy
0.32	0.33	104.3
0.64	0.62	97.2
1.6	1.64	102.6
3.2	3.24	101.4
8	7.90	98.8
16	15.67	97.9
40	38.64	96.6
80	79.88	99.9
200	203.47	101.7
400	398.35	99.6

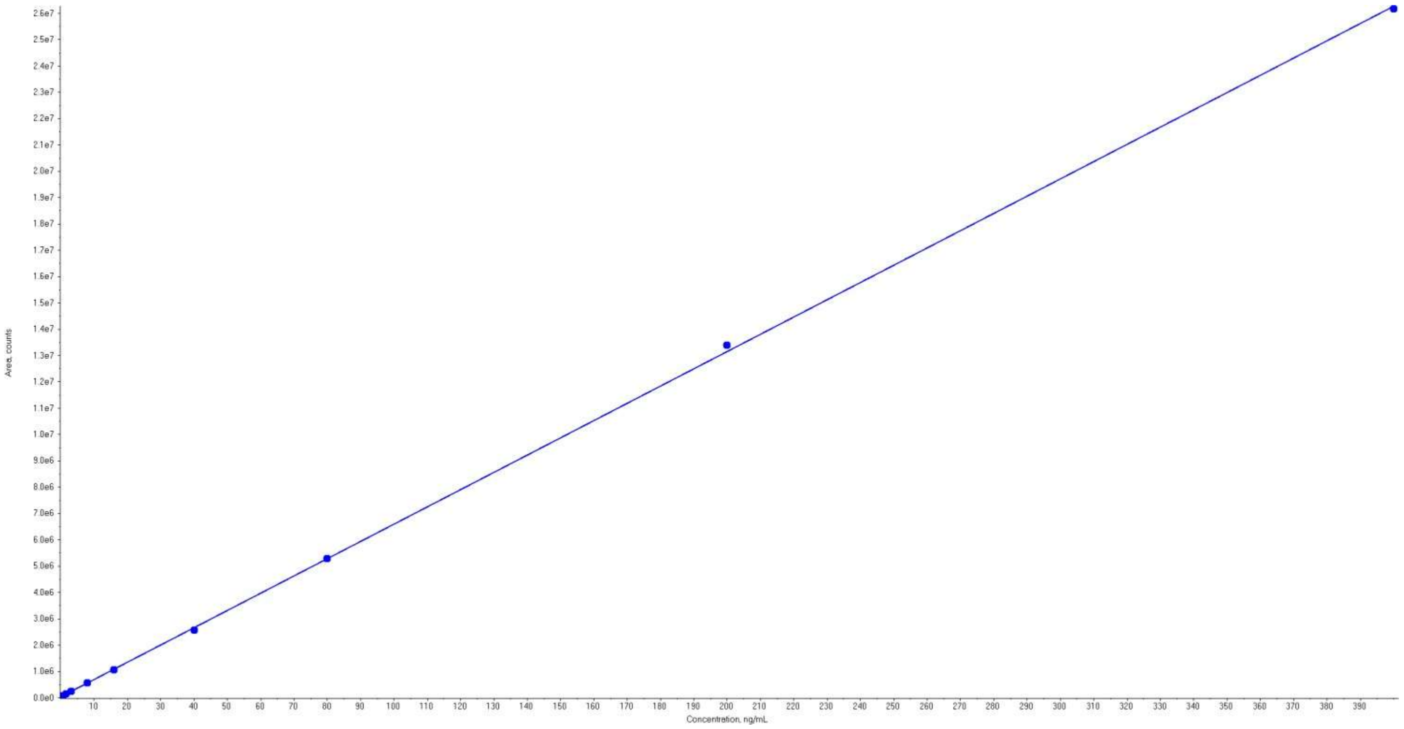

Analyte Name: Cordycepin

Regression Equation: y = 1.52e+004 x + 5.87e+004 (r = 0.9998)

Expected Concentration (ng/ml)	Mean Calculated Concentration (ng/ml)	% Accuracy
0.32	0.30	92.5
0.64	0.62	97.5
1.6	1.65	103.1
3.2	3.20	100.0
8	7.94	99.3
16	15.95	99.7
40	42.03	105.1
80	82.53	103.2
200	202.81	101.4
400	392.72	98.2

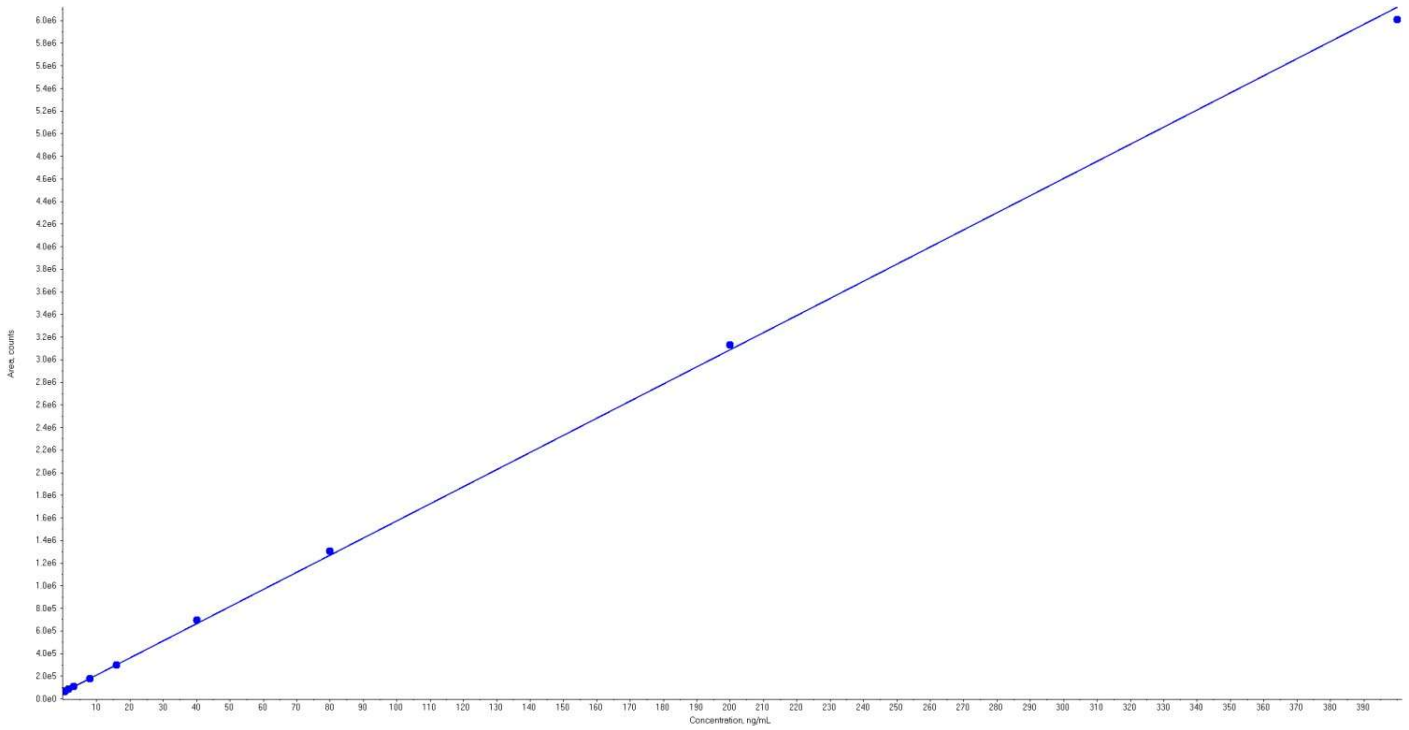

Results

Analyte Name	Spectrum	Calculated concentration (ng/ml)
Adenosine	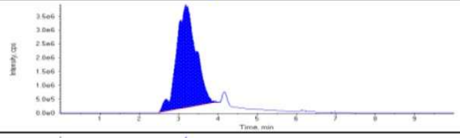	1960.0
Cordycepin	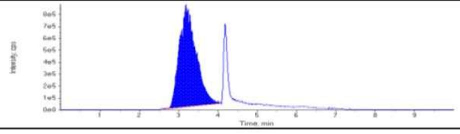	1620.0

Summary (The precise concentration of each metabolite in the sample; unit : ug/ml)

Calculation Formula: Analyte calculated concentration × 30 (dilution factor) / 1000

Analyte Name	Sample
Adenosine	58.80
Cordycepin	48.60
